# Evidence-informed health policy: are we beginning to get there at last?

**DOI:** 10.1186/1478-4505-7-30

**Published:** 2009-12-22

**Authors:** Stephen R Hanney, Miguel A González-Block

**Affiliations:** 1 Health Economics Research Group, Brunel University, Uxbridge, UK; 2 Center for Health Systems Research, National Institute of Public Health, Cuernavaca, Mexico

## The long-term challenge of increasing research use in health policymaking

Increasing the extent to which health policies are informed by health research has long been the hope, and indeed sometimes the expectation, of those reforming health research systems. Now though, there are grounds for believing that the hopes are increasingly beginning to be turned into realities. This optimism is based on a range of factors including: the growing understanding by researchers of the benefits of adopting a collaborative approach with policymakers in setting research agendas; the expansion of the pool of knowledge relevant for policymaking; the generation of capacity to conduct systematic reviews of that evidence; and the growing attention being given to the policymaking structures necessary to absorb and use research evidence. *Health Research Policy and Systems *(HARPS) has just published a supplement that draws together, and builds on, a very wide range of recent developments in this important field. Called *SUPPORT Tools for evidence-informed health Policymaking (STP)*, the supplement consists of a series of guides with an introduction setting out its scope and purpose [1]. Here we provide a brief analysis of why we think it is so appropriate to launch such an initiative at this time.

The Rothschild experiment that began in the UK in the early 1970s was one of the first attempts to create a system whereby a major part of the health research system would explicitly undertake projects that had been identified by policymakers as topics on which they wished to be informed by research. In 1983 Maurice Kogan and Mary Henkel published the results of a pioneering formative evaluation of this experiment [2]. Kogan and Henkel showed that for such an approach to be successful, a series of conditions would need to be met. They identified a key role for knowledge brokerage and policy analysis, they demonstrated how a government department could be seen as the receptor for research findings, and, above all, they highlighted the importance of collaboration between scientists and policymakers. In 2006 a second edition of the evaluation was published [3]. In commenting on the publication of the second edition, Jonathan Lomas said in relation to the original analysis:

Finally, the rest of the world has caught up with Kogan and Henkel. Twenty-five years ago their ground-breaking study of the UK's Department of Health led them to conclude that sustained interaction between scientists and bureaucrats was the key to unleashing the value of science for the policy process....They may have felt like voices in the wilderness then; today, however, they can take their rightful place as pre-cursors and leaders of what has become a mass-movement for 'evidence-based policy.' [4].

The importance of this historical perspective is that it helps to underline the difficulties, and complexities, of making progress in the field of greater exploitation of research or evidence in health policymaking. The obstacles facing the Rothschild experiment were such that rather than devoting more resources to building the capacity of the policymaking system to identify priorities and use the research, support was reduced and large parts of the experiment were abandoned [2,3].

## A wide range of initiatives

So, what is different now to suggest we can be more optimistic about the possibilities for research to make a greater impact on policymaking? In the intervening 25 years or so there have been many developments in this field. These include initiatives from the Research Policy and Cooperation Department at the World Health Organization (WHO). One such initiative was the establishment of HARPS, and the journal is increasingly providing a forum for articles, commentaries and discussion on the organisation of health research systems and the use of research to inform health policies. Hence, in this editorial our brief review of key developments in this field draws especially, but not exclusively, on articles published in HARPS.

In 2003 HARPS published a wide ranging analysis of developments in the research to heath policy field that partly built on the work of Kogan and Henkel and, for example, developed an 'interfaces and receptor model' for examining the impact of research on policy [5]. This review of impacts again demonstrates many of the difficulties described by others, but also suggests that perhaps there are more examples of research making an impact on policy, as broadly defined to include clinical policies, than are sometimes identified in other studies. This finding is further supported in a systematic review of attempts to assess the impact of health research programmes. The systematic review identifies a range of examples of research making some impact on health policy, again using a broad definition of policy [6]. It also indicates that when the assessment starts with research, and traces the impact forwards, it is more likely to identify impact than when the analysis starts with the policy and attempts to identify the impact made on it by research [6].

Even since that second review was published in 2007 there have been various further accounts of evidence-informed health policy, and these can now also be drawn upon to complement the expanding portfolio of examples. Recent examples reported in articles in HARPS include: the successful translation of research into maternal health care policy for the treatment of eclampsia and pre-eclampsia in South Africa [7]; evidence-based policymaking in the Philippines involving a randomised controlled policy experiment [8]; the long-term impact of health systems research on health reforms in Mexico [9]; and steps towards building equitable health systems in Sub-Saharan Africa through using operational research [10].

Various developments help account for the increasing use of research in health policymaking, despite the many continuing difficulties. There is a growing recognition of the importance of the collaborative approach between researchers and policymakers [11]. Under the leadership of Lomas, the Canadian Health Services Research Foundation (CHSRF) began putting the collaborative approach into practice using the 'Linkage and Exchange' concept [12]; and a widely cited review also highlighted the importance of interaction between researchers and policymakers [13]. The importance of brokerage roles is now much more widely acknowledged than it was at the time Kogan and Henkel demonstrated their importance in a health research system if research was to be used to inform health policymaking. Lomas and the CHSRF have played a key role in the last decade in demonstrating the value of knowledge brokers [14].

Most of the above discussion relates to systems where there are moves to increase research utilisation on health policymaking through the collaboration, in some form or another, between researchers and policymakers. But there have also been significant technical advances that encourage greater use of the global stock or pool of knowledge. The early 1990s saw the establishment of first the UK Cochrane Centre, and then the international Cochrane Collaboration, to conduct systematic reviews of clinical randomised controlled trials. The UK Cochrane Centre was supported by the National Health Service R&D Programme in the UK as part of its information systems strategy aimed at increasing the use of research findings [3]. Systematic reviews are now also increasingly covering healthcare management and policymaking [15].

The development of systematic reviews of the evidence has greatly facilitated the moves in health care towards developing guidelines, especially in clinical areas. In 2006 HARPS published a series of articles based on an initiative from the WHO's Advisory Committee on Health Research to identify ways in which WHO could improve the use of research evidence in guidelines. An introductory article in that series set out the full range of issues that were addressed in the 16 subsequent articles [16].

Furthermore, the development within some health care systems of various policymaking structures that act as receptor bodies for research, such as the National Institute for Health and Clinical Excellence (NICE) in the UK, has considerably extended the opportunities for research to be used in policymaking, and especially in clinical policymaking [6]. In many countries, however, where such structures are less developed or extensive, there are considerable barriers to developing evidence-based guidelines, as identified in the SEA-ORCHID project on the development of maternal and perinatal guideline development in hospitals in South East Asia [17].

So, there are many situations where the use of evidence in clinical policymaking is still very difficult. Furthermore, it has often proved even more challenging for research to make an impact on policies related to the organisation of the health care system, than it has been for research to impact on clinical matters [5]. Nevertheless, there are an increasing number of ways to encourage research use in health policymaking, as broadly defined. Many of these approaches have been incorporated, or analysed, in the increasingly wide ranging discussions on the best way of enhancing the use of research in health policy. Just a few of the many examples include: articles on ways to increase, and assess, the use of research findings in health policies [5,18,19] reports from international organisations such as the Alliance for Health Policy and Systems Research and the Council on Health Research for Development [20,21]; books on health policymaking [22,23]; and books on the use of evidence in policymaking in general [24]. There are also an increasing number of international initiatives aimed at increasing the use of research in policymaking about the organisation of health care systems. One such example, *Future Health Systems (FHS): Innovations for Equity*, is operating in six countries [25].

## Time to consolidate the progress made

On the one hand, therefore, there has been considerable progress towards evidence based policymaking in some systems, specific examples of research-informed policymaking can be identified, and there are many accounts of how greater use of research might be achieved. On the other hand, there are still many difficulties facing attempts to move towards evidence-informed health policymaking. As Kogan and Henkel identified, it is useful to address these issues using a systematic approach, and compared to the 1970s there is now much more experience to draw upon and many advances in the techniques that can be used. Therefore, it is now timely to publish the supplement containing the comprehensive series of practical guides called *SUPPORT Tools for evidence-informed health Policymaking (STP)*. They have been developed not only on the basis of previous literature, but also, at least in the case of some of the tools, through extensive application in the field and iterative adaption in the light of these experiences. The guides cover a wide spectrum of issues related to supporting evidence-informed health policymaking and we anticipate that they will be a major, practical contribution to advancing this field.
